# Nanoemulsions for Skin Delivery of Essential Oils: A Systematic Review

**DOI:** 10.1002/ptr.70184

**Published:** 2026-01-10

**Authors:** Thais Leticia Moreira da Silva, Anna Claudia Morais de Oliveira Capote, Flávio Luís Beltrame, Priscileila Colerato Ferrari

**Affiliations:** ^1^ Pharmaceutical Science Post‐Graduation Program State University of Ponta Grossa Ponta Grossa Paraná Brazil; ^2^ Department of Pharmaceutical Sciences State University of Ponta Grossa Ponta Grossa Paraná Brazil

**Keywords:** dermatology, nanocarriers, nanoencapsulation, nanotechnology, skin permeation, volatile oil

## Abstract

Essential oils (EOs) are recognized for their multiple health benefits. However, their high volatility, low stability, and limited water solubility limit their effective application. This systematic review aims to assess the use of nanoemulsions as delivery systems for the topical administration of EOs, highlighting their efficacy, safety, and limitations. A literature search was conducted in the PubMed, Scopus, and Web of Science databases for studies published in English before February 2025, following the PRISMA 2020 guidelines. Studies limited to in vitro or ex vivo assays, using isolated EO components, or involving non‐topical applications were excluded. Twenty‐two articles were included in this review, comprising EOs from 18 plant species, and applied in animal or human in vivo models for wound healing (*n* = 6), anti‐inflammatory/analgesic effects (*n* = 5), cosmetic (*n* = 6), and transdermal delivery/permeation enhancer (*n* = 5). Nanoemulsions improved EOs' bioactivities, particularly their anti‐inflammatory, antioxidant, and antimicrobial effects, by enhancing skin permeation, bioavailability, and skin barrier function, reducing skin irritation, and allowing a controlled release. However, the overall risk of bias, assessed using the SYRCLE and RoB 2 tools, was considered high, and the studies' heterogeneity limited direct comparisons. Therefore, further well‐designed preclinical and clinical trials are needed to validate these findings and assess the potential of the EOs nanoemulsions for topical use.

**Abbreviations**
DNFB: 2,4 dinitrofluorobenzene
DPPH: 1,1‐diphenyl‐2‐picrylhydrazyl
EO: essential oil
GRAS: generally recognized as safe
HaCaT: human keratinocyte cell line
IgE: immunoglobulin E
IL: interleukin
MIB: minimum bactericidal concentration
MIC: minimum inhibitory concentration
NF‐κB: nuclear factor‐kappa B
NLCs: nanostructured lipid carriers
NO: nitric oxide
PASI: psoriasis area and severity index
PEG: polyethylene glycol
PGE2: prostaglandin E2
PVA: polyvinyl acetate
SLNs: solid lipid nanoparticles
SPF: sun protection factor
TEWL: transepidermal water loss
TNF: tumor necrosis factor‐alpha
UVB: ultraviolet B
VEGF: vascular endothelial growth factor

## 1 Introduction

Plant species can produce a wide range of products that humans have historically utilized, with essential oils (EOs) being one of the most well‐known. EOs are a complex mixture of volatile, lipophilic, and odoriferous substances (terpene hydrocarbons, simple alcohols, aldehydes, ketones, phenols, esters, furans, organic acids, lactones, coumarins, and others) commonly found in aromatic plants. These natural products are typically colorless or pale yellow, liquid at room temperature, and less dense than water (Sousa et al. 2023). Their chemical constituents have a low molecular weight (< 300), and the specific composition of each EO varies in quality and quantity depending on the plant species from which they are extracted, plant part, growing conditions, and other environmental factors (Sousa et al. 2023; Bunse et al. 2022).

EOs have gained significant popularity in various industries, including aromatherapy, food flavoring, and natural pharmacological treatments, due to their primary compounds and respective biological properties (Pezantes‐Orellana et al. 2024). Generally, two or three constituents are present in EOs at higher concentrations (20%–95%) and are typically responsible for their characteristic odor and bioactivity. However, minor constituents (1%–20%) and trace constituents (< 1%) also play an important role either by potentiating the action or through antagonistic or additive effects (Sousa et al. 2023; Pezantes‐Orellana et al. 2024; Heinzmann et al. 2017).

Numerous studies have focused on the applications of EO, exploring its antimicrobial, anti‐inflammatory, analgesic, and antioxidant properties, among others (Pezantes‐Orellana et al. 2024). The therapeutic effects of EOs are attributed to their distinct routes of administration. One of the most common methods is topical and transdermal application, through which their constituents can penetrate various skin layers, mucous membranes, muscles, and joints, offering an innovative route in the herbal drug delivery system and providing tremendous advantages compared to oral administration (Bunse et al. 2022; Aziz et al. 2019). These benefits are attributed mainly to terpenes and their oxygenated derivatives, terpenoids, the primary constituents of OEs, which are efficient, safe, and natural lipophilic compounds capable of interacting with stratum corneum lipids (the outermost layer of the skin) and permeating deeper skin layers. Then, they can be applied topically for local action, as well as to reach dermal or even systemic targets (Pezantes‐Orellana et al. 2024; Lasoń 2020).

However, some factors limit the pharmacological application of EOs, including their high volatility and lipophilicity (water insolubility), susceptibility to degradation, and sensitivity to environmental variables (Bahloul et al. 2024). Additionally, direct exposure to the skin is not recommended due to the risk of an allergic reaction (Kazemi et al. 2020).

To overcome these limitations, EOs have been loaded into nanoparticles using nanoencapsulation technology (Bahloul et al. 2024). Nanotechnology presents a promising approach, as it enables the manipulation of variables such as the type, size, and charge of nanoparticles, as well as their biochemical properties, including hydrophobicity and interaction with biological targets. This enables improvements and facilitates tissue penetration, promotes cellular interaction, and accelerates the accumulation process in various cell types (Bahloul et al. 2024; Fakhraei et al. 2025).

A nanoemulsion system is an advanced nanocarrier designed for high‐performance transdermal applications, capable of incorporating a variety of lipophilic compounds. With droplet sizes ranging from 10 to 200 nm, this system offers excellent kinetic, physicochemical, and thermodynamic stability. It also enhances bioavailability and effectively protects lipophilic substances and their components (Flekka et al. 2024; Miastkowska et al. 2023; Morteza‐Semnani et al. 2022).

Therefore, the number of studies proposing the encapsulation of EOs for application in the most diverse areas is increasing and has been well documented in scientific literature. Thus, this systematic review provides a comprehensive overview of the potential applications of EO nanoemulsions and evaluates their effects on animal and human skin, assessing efficacy, safety, permeation capacity, and pharmacological properties. Furthermore, the review discusses preparation methods, formulation components, characterization techniques, and key findings from these studies, highlighting both the advantages and challenges of using nanoemulsions as topical delivery systems for EOs.

## 2 Methods

### 2.1 Search Strategy

This systematic review was conducted following the preferred reporting items for systematic reviews and meta‐analyses (PRISMA) guidelines (review not registered) (Table S1). The literature search was conducted in February 2025 by two independent authors (TLMS and ACMOC) using the scientific online databases PubMed, Scopus, and Web of Science (core collection), with the key terms: “nanoemulsion” AND “essential oil” AND “skin.” No filters were applied. Additionally, the reference lists of the selected articles and relevant reviews were screened to identify additional eligible studies not retrieved by the primary search.

### 2.2 Study Selection

Titles were screened, and duplicate records were removed. Then, review papers were excluded, and the abstracts of the remaining studies were screened. Only full‐text original articles published in English were considered eligible. Full‐text articles were reviewed to determine eligibility for inclusion based on the inclusion and exclusion criteria. The inclusion criteria were: (1) studies that formulated and investigated the application of nanoemulsions or nanoemulsion‐based products; (2) application of EO as an active ingredient; (3) studies with in vivo tests using animal or human skin. The exclusion criteria involved: (1) studies limited to in vitro and/or ex vivo tests; (2) isolated EO components as active ingredients; (3) studies about food applications, oral administration, and other non‐topical applications.

### 2.3 Data Extraction

Data from each study included in this review were manually extracted from the abstract, main text, tables, figures, and Supporting Information by one author (TLMS) and reviewed by another author (ACMOC) to minimize the risk of bias. The extracted data can be summarized as: (1) common name and species of the plant from which the EO was obtained; (2) aim of the research; (3) nanoemulsions formulation (surfactant and/or co‐surfactant, other ingredients used and their respective functions, each component concentration and nanoemulsification technique); (4) nanoemulsion characterization (average droplet size, polydispersity index (PDI), and zeta potential); (5) in vitro, ex vivo and in vivo studies (methodology, control groups and key findings); (6) primary outcomes (advantages and disadvantages of nanotechnology used); (7) statistical analysis. If some data were not in the same unit of measurement as in other articles or were unavailable, data conversions were performed. All collected data were tabulated into a predefined table using Microsoft Excel 365 version 2501.

### 2.4 Risk of Bias

The Systematic Review Center for Laboratory Animal Experimentation (SYRCLE) checklist (Hooijmans et al. 2014), comprising 10 items, was used to assess the quality and risk of bias in the in vivo study. Two reviewers independently analyzed each study and assigned a risk level to each item, categorizing it as high risk, low risk, or moderate (Figure S1). Four studies involving human participants were conducted, and the risk of bias in these studies was assessed using the cochrane risk of bias (RoB 2) tool (Sterne et al. 2019) (Figure S2).

## 3 Results

### 3.1 Selected Studies

**FIGURE 1:**
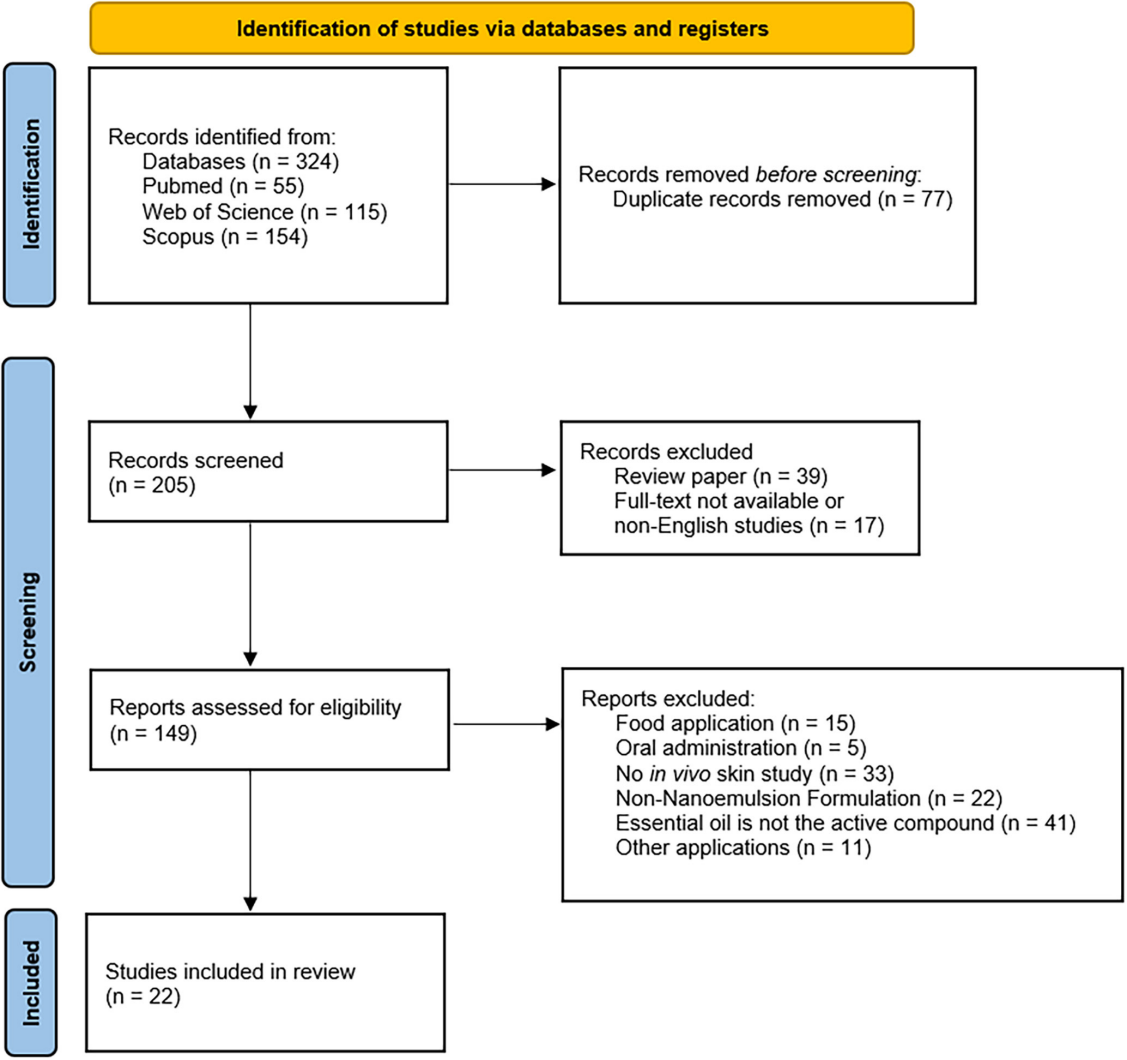
PRISMA flow diagram.

Initially, 324 records were identified through the database search. After the removal of duplicates (*n* = 77) and ineligible records (*n* = 56), 149 articles were fully screened. No additional eligible studies were identified in the reference lists of the selected articles beyond those already found in the initial search. The final systematic review resulted in 22 articles that met all the inclusion and exclusion criteria, which are summarized in a PRISMA flow diagram (Figure 1).

### 3.2 Characteristics of Included Studies

Considering the heterogeneity between studies regarding the EO used, nanoemulsion formulations, topical application objectives, and the type of tests performed, each study is individually described and discussed in detail in the following sections. The selected studies are organized based on the formulation characteristics (Table 1), nanoemulsion characterization (Table 2), and in vivo tests involving the final product application (Table 3).

**TABLE 1:** Characteristics of nanoemulsion formulations from the included studies.

Essential oils		Surfactant		Co‐surfactant		Technique	Final product	Thickener	References
Plant	%	Name	%	Name	%				
*Eucalyptus globulus* (eucalyptus)	16.66	Tween 80	16.66			Ultrasonication	Nanoemulsion		Guerrero et al. (2024)
*Melaleuca alternifolia* (tea tree)	5	Tween 20	6	Cremophor EL	10	High‐speed homogenization	Nanoemulgel	Carbopol 940	Kang et al. (2024)
*Eucalyptus globulus* (eucalyptus)	2.5	Tween 40	3–18			Spontaneous emulsification	Nanoemulsion		Aziz et al. (2019)
*Eucalyptus globulus* (eucalyptus)	2.5	Tween 60	12–18			Spontaneous emulsification	Nanoemulsion		Aziz et al. (2019)
*Eucalyptus globulus* (eucalyptus)	2.5	Tween 80	12–18			Spontaneous emulsification	Nanoemulsion		Aziz et al. (2019)
*Lavandula angustifolia* Mill (lavender)	2	Tween 20/Tween 80 (2:1)	7.5			Spontaneous emulsification	Nanoemulsion‐based cream	Eucerin cream	Kazemi et al. (2020)
*Rosmarinus officinalis* L. (rosemary) and *Mentha piperita* (peppermint)	—	Tween 20/Tween 80 (1:3)	—			Spontaneous emulsification	Nanoemulsion		Mohammadifar et al. (2021)
*Allium sativum* (garlic) and *Zingiber officinale* (ginger)	0.1	Tween 80	—			Ultrasonication	Nanoemulsion		Ibrar et al. (2022)
*Cuminum cyminum L*. (cumin)	2–4	Tween 80/Span 80 (1:1)	3			High‐pressure homogenization	Nanoemulgel	Carbopol 940	Morteza‐Semnani et al. (2022)
*Pelargonium graveolens* (geranium)	27.5	Tween 80/Span 80 (HLB 12)	60	Transcutol	—	Spontaneous emulsification	Nanoemulsion		Infante, Darvin, and Campos (2023)
Eucalyptus (species not reported)	15	Tween 20	26.25	Span 80	8.75	High‐speed homogenization	Nanoemulgel	Carbopol 934 and hydroxypropyl methylcellulose	Xiao et al. (2021)
Clove (species not reported)	20	Tween 20	33.75	Span 80	11.25	High‐speed homogenization	Nanoemulgel	Carbopol 934 and hydroxypropyl methylcellulose	Xiao et al. (2021)
*Aniba canelilla* (Kunth) Mez (precious bark)	10	Tween 20/Span 80 (1:2)	3			High‐pressure homogenization	Hydrogel‐thickened nanoemulsion	Hydroxyethylcellulose and chitosan	Mssillou et al. (2025)
*Lavandula angustifolia* Mill (lavender)	1	Arlacel 2121	2.5			Ultrasonication	Nanoemulsion		Miastkowska et al. (2023)
*Pituranthos tortuosus* (Coss.) Maire	1	Tween 80	14.4	Span 80	5.6	High‐speed homogenization	Nanoemulgel	Polyacrylate crosspolymer‐6	Bahloul et al. (2024)
Sweet fennel (species not reported)	5	Cremophor RH40	6			Ultrasonication	Nanoemulsion		Chaurasiya et al. (2024)
*Artemisia monosperma*	1	Tween 80	5	Ethanol	1	Phase inversion	Nanoemulsion		Tawfik et al. (2024)
*Matricaria chamomilla* (chamomile)	—	Polyoxyethylenated castor oil (EL‐40)	16	Ethanol	5.4	Spontaneous emulsification	Nanoemulgel	*Bletilla striata* polysaccharide	Kreutz et al. (2023)
*Rosmarinus officinalis* L. (rosemary)	10	Cremophor EL	50	Labrafil	20	Spontaneous emulsification	Nanoemulgel	Carbopol 934	Abla et al. (2025)
*Perovskia abrotenoides* Karel	5	Tween 80	36	Ethanol	9	Ultrasonication	Hydrogel‐thickened nanoemulsion	Chitosan	Fakhraei et al. (2025)
*Rosa damascena*	2	Tween 20/Tween 80 (1:1)	4			Spontaneous emulsification	Nanoemulgel	Carboxymethyl cellulose	Infante, Campos, et al. (2023)
*Melaleuca alternifolia* (tea tree)	2	Polyglyceryl‐6 distearate	3			Not specified	Nanoemulsion		Pandey et al. (2024)
*Melaleuca alternifolia* (tea tree)	2	Polyglyceryl‐6 distearate	3			Not specified	Nanoemulsion		Nhani et al. (2024)
Peppermint and myrtile	5–10	Tween 80/propylene glycol (1:0.75)	4			Microwave‐based method	Nanoemulgel	Carbopol 940	Drais (2023)
*Lavandula angustifolia* Mill (lavender)	—	Lecithin and PEG‐15‐hydroxystearate	—			Ultrasonication	Nanoemulsion		Flekka et al. (2024)

**TABLE 2:** Charactherization of the nanoemulsions in terms of droplet size, polydispersity index (PDI) and zeta potential.

Essential oil	Average droplet size (nm)	PDI	Zeta potential (mV)	References
Eucalyptus globulus (eucalyptus)	3.8			Guerrero et al. (2024)
Melaleuca alternifolia (tea tree)	16	0.412–0.583	36	Kang et al. (2024)
Eucalyptus globulus (eucalyptus)	12.57–42.85	0.222–0.495		Aziz et al. (2019)
Eucalyptus globulus (eucalyptus)	54.07–72.97	0.861–0.935		Aziz et al. (2019)
Eucalyptus globulus (eucalyptus)	23.51–45.01	0.548–0.796		Aziz et al. (2019)
Allium sativum (garlic) and Zingiber officinale (ginger)	145–304	0.097–0.349	−3.0–1.57	Ibrar et al. (2022)
Cuminum cyminum L. (cumin)	82.20–187.53	0.200–0.240	−0.50–3.04	Morteza‐Semnani et al. (2022)
Pelargonium graveolens (geranium)	61–138	0.090–0.400	−17.3	Infante, Darvin, and Campos (2023)
Eucalyptus	78	0.260	−22	Xiao et al. (2021)
Clove	67	0.240	−19	Xiao et al. (2021)
Aniba canelilla (Kunth) Mez (precious bark)	100–150	0.210	−36	Mssillou et al. (2025)
Lavandula angustifolia Mill (lavender)	187	0.178		Miastkowska et al. (2023)
Pituranthos tortuosus (Coss.) Maire	27	0.300	−22.8	Bahloul et al. (2024)
Sweet fennel	21.12	0.159		Chaurasiya et al. (2024)
Artemisia monosperma	228	0.406	−9.4	Tawfik et al. (2024)
Matricaria chamomilla (chamomile)	19.07		−0.237	Kreutz et al. (2023)
Rosmarinus officinalis (rosemary)	125–252	0.103–0.325	−19.9	Abla et al. (2025)
Perovskia abrotenoides Karel	13.2	0.203	−2.9	Fakhraei et al. (2025)
Rosa damascena	86		−46	Infante, Campos, et al. (2023)
Peppermint and myrtile	25.83–49.83	0.260–0.385	12.61–19.60	Drais (2023)
Lavandula angustifolia Mill (lavender)	101.63	0.230	−71.4	Flekka et al. (2024)

**TABLE 3:** Summary of in vivo studies on the topical application of essential oil nanoemulsions.

Essential oils	In vivo study	In vivo model	Nanoemulsion results	References
*Eucalyptus globulus* (eucalyptus)	Skin irritation	Male Wistar albino rats (*n* = 12)	No skin irritation.	Guerrero et al. (2024)
	Wound healing	Male Wistar albino rats (*n* = 16)	Complete wound healing (100%) by day 16, outperforming non‐treated and positive control (neomycin) groups (94.2%).	
*Lavandula angustifolia* Mill (lavender)	Wound healing	Wistar albino rats (*n* = 85)	Showed higher wound healing by day 14 compared to conventional emulsion, non‐treated, positive control (phenytoin 1% ointment) and placebo (Eucerin ointment) groups. Up‐regulated the expression of TGF‐β1 and collagen I/III genes.	Kazemi et al. (2020)
*Lavandula angustifolia* Mill (lavender)	Skin irritation	New Zealand albino rabbits (*n* = 3)	No skin irritation.	Miastkowska et al. (2023)
	Skin sensitization	Guinea pigs (*n* = 18)	Concentrations above 50% up to 100% caused moderate erythema in pigs.	
*Pituranthos tortuosus* (Coss.) Maire	Wound healing	Wistar rats (*n* = 20)	Complete wound healing by day 10, outperforming non‐treated, conventional emulsion‐gel, negative control (blank formulation) and positive control (MEBO) groups.	Bahloul et al. (2024)
Sweet fennel (species not reported)	Wound healing	C57BL/6 mice (*n* = 8)	Showed higher wound healing by day 7 compared negative control (saline solution) group.	Chaurasiya et al. (2024)
*Perovskia abrotenoides* Karel	Wound healing	Female Wistar rats (quantity not specified)	Incorporation into chitosan showed higher wound healing by day 21 (95%), compared to simple nanoemulsion (77.7%), non‐treated (60%), positive control (phenytoin cream) (90%) and negative control (chitosal gel 2%) (85%) groups.	Fakhraei et al. (2025)
*Eucalyptus globulus* (eucalyptus)	Hot plate test	Male Sprague Dawley (*n* = 18)	Slightly prolonged and sustained analgesic activity compared to pure EO and significantly prolonged it compared to negative control (saline solution), demonstrating an enhancement of analgesic activity.	Aziz et al. (2019)
*Mentha piperita* (peppermint) and *Rosmarinus officinalis* L. (rosemary)	Skin irritation	New Zealand white rabbits (*n* = 3)	No skin irritation.	Mohammadifar et al. (2021)
	Acute dermal toxicity	New Zealand white rabbits (*n* = 6)	After 14 days, no abnormality was observed in the skin, eyes, mucous membranes, and behavioral patterns.	
	Behavioral studies after induction of osteoarthritis	Wistar rats (*n* = 36)	Decreased the mechanical and thermal allodynia, thermal hyperalgesia, and ambulatory‐evoked pain via increasing SOD and GPx activity, decreasing MDA levels, and improving the pathological features of rats' knee joint with osteoarthritis.	
*Aniba canelilla* (Kunth) Mez (precious bark)	Anti‐inflammatory potential in croton oil‐induced mice ear edema	Male CF‐1 mice (~*n* = 42)	Edema inhibition was statistically equivalent between pure EO (37.64%), nanoemulsion (37.40%) and hydrogel nanoemulsion (36.24%) groups, and higher than blank formulations (14.04%).	Mssillou et al. (2025)
*Artemisia monosperma*	Anti‐psoriatic effect using imiquimod to induce a psoriasis‐like dermatitis model	BALB/c mice (*n* = 30)	Effectively decreased the PASI score and rat spleen weight, inhibited the inflammatory mediators TNF‐α, IL‐6, and IL‐17, decreased inflammatory cell infiltration, and attenuated epidermal thickening and hyperkeratosis.	Tawfik et al. (2024)
*Matricaria chamomilla* (chamomile)	Treatment of atopic dermatitis induced by 2,4 dinitrofluorobenzene (DNFB)	Male BALB/c mice (*n* = 25)	Reduced epidermal thickness, mast cell infiltration, IgE, and inflammatory cytokines production, outperforming pure EO and being comparable to positive control (dexamethasone).	Kreutz et al. (2023)
	Topical safety by determining treated mice hepatorenal function	Male BALB/c mice (*n* = 5)	No significant changes in hepatic enzymes, creatinine, or urea nitrogen levels, indicating hepatic and renal safety by topical administration.	
*Melaleuca alternifolia* (tea tree)	Skin irritation	New Zealand white rabbits (*n* = 6)	No skin irritation.	Kang et al. (2024)
*Rosa damascena*	Treatment of wrinkles induced by UVB radiation	Male Wistar rats (*n* = 26)	Application immediately after UVB exposure reduces wrinkle formation on rats' skin. Pre‐treatment was not effective.	Infante, Campos, et al. (2023)
*Melaleuca alternifolia* (tea tree)	Clinical evaluation of facial skin characteristics over 90 days	Male participants (*n* = 40) aged between 18 and 28 years	Increased the *stratum granulosum* keratinocyte area and improved its morphology, reduced *stratum corneum* thickness and skin surface sebum, increased hydration and collagen fiber density in the papillary dermis. Reached deeper layers of the skin compared to pure EO.	Pandey et al. (2024)
*Melaleuca alternifolia* (tea tree)	Clinical evaluation of facial non‐inflammatory acne over 90 days	Male participants (*n* = 53) aged between 18 and 28 years	Improved the morphological and structural characteristics associated with comedones within the pilosebaceous units. Reached deeper layers of the skin compared to pure EO.	Nhani et al. (2024)
Peppermint and myrtile	Skin irritation test and sensory analysis	Human volunteers (*n* = 30)	No skin irritation. Formulations were rated as comfortable and well‐tolerated regarding texture, appearance, smell, redness, and irritation or burning sensation.	Drais (2023)
*Lavandula angustifolia* Mill (lavender)	Clinical evaluation of skin characteristics before and after a single application	Men and women (*n* = 10) aged between 20 and 60 years.	Increased skin hydration compared to baseline, sustaining it from 30 min to 2 h post‐application. Reduced transepidermal water loss (TEWL) compared to untreated skin, indicating accelerated barrier repair.	Flekka et al. (2024)
*Allium sativum* (garlic) and *Zingiber officinale* (ginger)	Wound healing	Male and female rabbits (*n* = 36)	The EOs nanoemulsion containing neomycin sulfate promoted complete wound healing (100%) by day 9, outperforming pure neomycin sulfate (71%).	Ibrar et al. (2022)
*Cuminum cyminum* L. (cumin)	Rat tail‐flick	Male NMRI mice (*n* = 6)	All formulations containing diclofenac showed analgesic activity.	Morteza‐Semnani et al. (2022)
	Formalin test on mouse paw (licking time) for pain response	Male Wistar rats (*n* = 6)	However, the nanoemulgel significantly prolonged pain tolerance and enhanced analgesic effects.	
*Pelargonium graveolens* (geranium)	Burn wound healing	Rats (*n* = 84)	A synergistic effect between geranium EO and pravastatin was observed, as higher concentrations of both actives reduced burn wound diameter and the IL‐6 serum level.	Infante, Darvin, and Campos (2023)
Clove and eucalyptus (species not reported)	Skin irritation	Male Wistar rats (*n* = 30)	No skin irritation.	Xiao et al. (2021)
*Rosmarinus officinalis* L. (rosemary)	Hair growth effect	Wistar albino rats (*n* = 50)	A synergistic effect between rosemary EO and metformin was observed, as it accelerated hair follicle growth compared to blank formulations and positive control (minoxidil).	Abla et al. (2025)

All selected studies focused on developing nanoemulsions to enhance the physicochemical stability of EOs and improve their therapeutic activity, permeation, or retention in the skin layers. Most of them evaluated the antimicrobial and anti‐inflammatory properties of EO nanoemulsions, highlighting their role in promoting wound healing, reducing the risk of skin infections, and enhancing the topical delivery of other drugs.

This review includes only studies that investigated the direct application of nanoemulsions on animal or human skin through in vivo experiments. Eighteen studies conducted in vivo experiments using animals (rats (*n* = 14), rabbits (*n* = 4), or guinea pigs (*n* = 1)), and 4 in vivo studies used human volunteers. Some in vitro and ex vivo tests, such as antimicrobial, antioxidant, cytotoxicity, and permeation/retention using synthetic membranes or animal skin, are also described. The most tested microorganisms were *Staphylococcus aureus* (*n* = 7), *Escherichia coli* (*n* = 4), *Pseudomonas aeruginosa* (*n* = 2), and *Candida albicans* (*n* = 3), along with *Streptococcus mutans* , *Staphylococcus epidermidis* , *Propionibacterium acnes* , *Bacillus spizizenii*, and *Salmonella enterica* .

### 3.3 Essential Oils Used and Their Role in Formulations

The studies included 18 EOs from different plants. The most used were *Eucalyptus globulus* (eucalyptus) (*n* = 3), *Lavandula angustifolia* Mill (lavender), and *Melaleuca alternifolia* (tea tree) (*n* = 3). *Mentha piperita* (peppermint) and *Rosmarinus officinalis* (rosemary) were tested in two studies each (*n* = 2). Other EOs studied include *Allium sativum* (garlic), *Zingiber officinale* (ginger), *Aniba canelilla* (precious bark), *Artemisia monosperma*, *Cuminum cyminum* (cumin), sweet fennel (species not specified), *Eugenia caryophyllus* (clove), *Matricaria chamomilla* (chamomile), *Myrtus* sp. (myrtle), *Pelargonium graveolens* (geranium), *Perovskia abrotanoides*, *Pituranthos tortuosus*, and *Rosa damascena*. In most studies, the EO serves as an active formulation agent responsible for the therapeutic activity investigated; however, five studies utilized the EO in combination with other drugs as permeation enhancers to achieve a synergistic effect (Table 3).

### 3.4 Nanoemulsion Formulation

The studies included four types of nanoemulsion formulations (nanoemulsion, nanoemulsion‐based gel (nanoemulgel), hydrogel‐thickened nanoemulsion, and nanoemulsion‐based cream) and six nanoemulsion techniques (spontaneous emulsification, ultrasonication, high‐pressure homogenization, high‐speed homogenization, microwave‐based method, and phase inversion).

**FIGURE 2:**
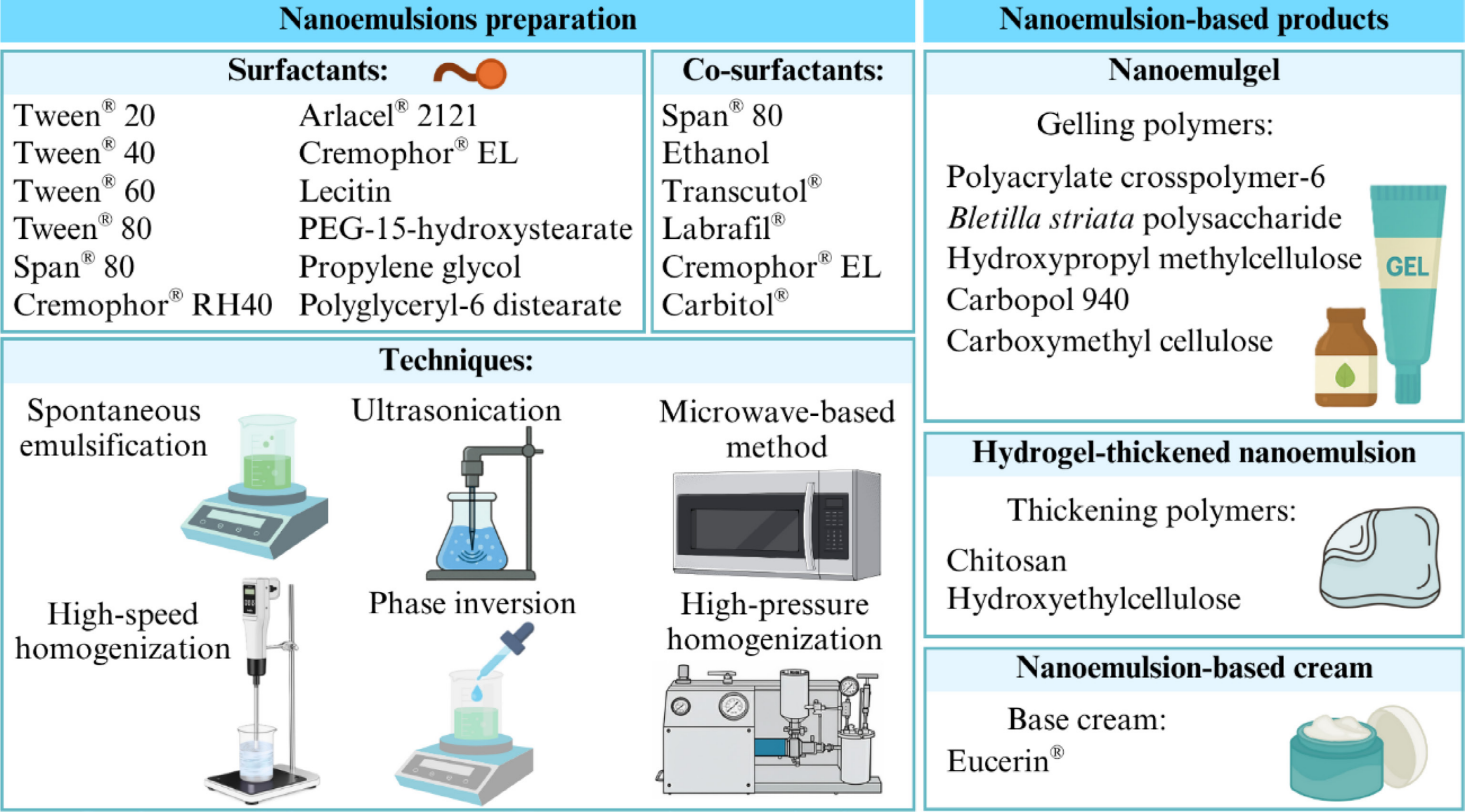
Overview of components (surfactants and co‐surfactants) and techniques for EO nanoemulsion preparation, and nanoemulsion‐based final products.

The most commonly used surfactants to produce nanoemulsions were Tween 80 (*n* = 13), Tween 20 (*n* = 7), Span 80 (*n* = 3) and polyglyceryl‐6 distearate (*n* = 2), followed by Cremophor RH40, Arlacel 2121, Cremophor EL, Lecitin, PEG‐15‐hydroxystearate, Polyoxyethylenated castor oil (EL‐40), Tween 40, Tween 60 and propylene glycol. Seven studies used a combination of two surfactants, and 13 studies employed co‐surfactants, including Span 80, ethanol, Transcutol, Labrafil, Cremophor EL, and Carbitol (Figure 2).

The concentration of EO in the formulations ranged from 0.1% to 27.5%. Table 1 presents the nanoemulsion components and detailed characteristics of the formulations. Nanoemulsions characterization in terms of droplet size, PDI, and zeta potential is presented in Table 2.

None of the included studies investigated potential correlations between the physicochemical properties of the nanoemulsions and the observed biological effects. Consequently, these parameters do not appear to impact the biological activity significantly. However, the type of nanoemulsion formulation was found to be relevant to the outcomes. Therefore, the present study focused on the nanotechnological approach, rather than minor variations in physicochemical characteristics.

### 3.5 Risk of Bias

To minimize bias and prevent underestimating or overestimating results, experimental tests and measurements should be randomized and blinded. However, these practices are rarely reported in animal studies. This was clearly observed in this review, as most animal studies were classified as having a high risk of bias (*n* = 11), primarily due to the absence of allocation concealment, randomization, and blinding. Moreover, although some authors mentioned the allocation and outcome assessment methodology, their studies were categorized as having a moderate risk of bias (*n* = 8) due to insufficiently reported experimental details. Only one animal study was classified as having a low risk of bias. In contrast, the studies in humans were mainly classified as having a low risk of bias, except for Drais (Drais 2023), whose study was categorized as having a moderate to high risk of bias.

## 4 Discussion

### 4.1 Nanoemulsions

Nanoemulsions are kinetically stable dispersions of oil‐in‐water or water‐in‐oil, stabilized by an interfacial layer of surfactants, often combined with a co‐surfactant, forming droplets with diameters smaller than 200 nm (Figure 2) (da Silva et al. 2019; Mohammadifar et al. 2021). Their nanometric size, significantly smaller than the wavelength of visible light, makes nanoemulsion systems appear transparent or translucent (da Silva et al. 2019). Unlike conventional emulsions, their reduced droplet size and steric stabilization, achieved through appropriate surface functionalization, confer long‐term stability against creaming, sedimentation, aggregation, flocculation, coalescence, and Ostwald ripening (Flekka et al. 2024; Mostafa et al. 2015).

To develop a nanoemulsion, information about the physicochemical properties of the active ingredient and its intended application is necessary to select the appropriate surfactant, as it affects the droplet size, density, viscosity, interfacial tension, and phase behavior (Kakadia and Conway 2022). Among various options, non‐ionic surfactants such as Tweens and Spans are preferred for therapeutic purposes, due to their ability to spontaneously form micelles at the oil–water interface, reduced toxicity, and greater stability against changes in pH (Aziz et al. 2019; Kakadia and Conway 2022). This preference is supported by the findings of the present review, in which Tween was the surfactant employed in 15 out of the 22 selected studies, with Span additionally incorporated in 6 of these formulations.

In addition to surfactant selection, the method of preparation plays a critical role in determining the final characteristics of the nanoemulsion and must be considered (Figure 2). To reduce micelle size, high‐energy methods (ultrasonication, microwave‐based techniques, high‐pressure or high‐speed homogenization) are often required. These techniques utilize mechanical devices to generate strong disruptive forces capable of producing tiny oil droplets, which leads to increased stability and surface area. However, since high‐energy methods require specialized equipment, low‐energy methods (such as spontaneous emulsification, solvent displacement, and phase inversion) have also been explored, which are more straightforward, cost‐effective, and gentler on sensitive ingredients; however, they may result in larger droplet sizes (Chavda et al. 2024).

**FIGURE 3:**
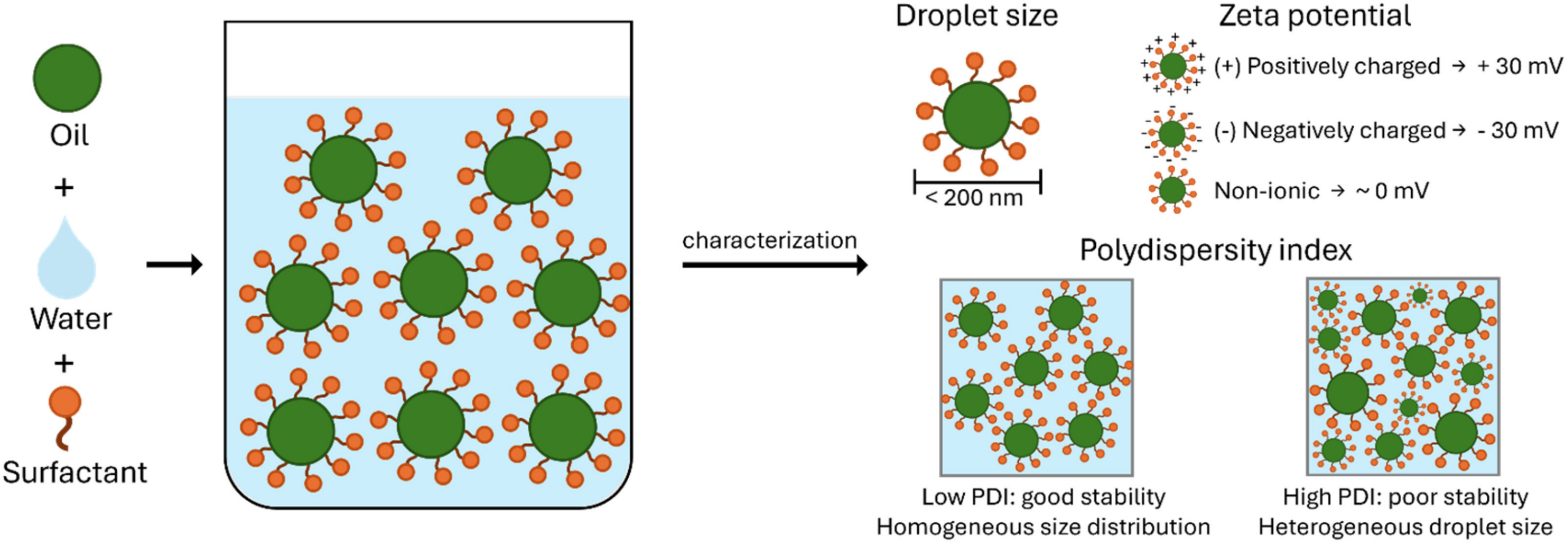
Schematic representation of an oil‐in‐water nanoemulsion system and the ideal physicochemical characteristics of the formulation required to ensure quality and stability.

Therefore, characterization of nanoemulsions is essential to confirm the adequacy of the formulation ingredients and method of preparation. Droplet size, polydispersity index (PDI), and zeta potential are the primary physicochemical parameters analyzed to evaluate the quality, stability, and suitability of nanoemulsions for their intended application (Figure 3). The results of these analyses performed by the studies included in this review are presented in Table 2.

A smaller droplet size (< 200 nm) contributes to improved EOs bioavailability by enabling penetration into deeper layers of the skin, increasing the solubility of lipophilic agents, and offering kinetic stability, which prevents gravitational separation and droplet aggregation (Bahloul et al. 2024; Mohammadifar et al. 2021). The PDI is a major predictor of droplet size homogeneity and is generally used to evaluate the stability of nanoemulsions. Higher PDI significantly increases the likelihood of Ostwald ripening, one of the primary mechanisms of nanoemulsion instability (Morteza‐Semnani et al. 2022; Tawfik et al. 2024). In contrast, lower PDI indicates a homogeneous size distribution, which enhances formulation stability and reliability, enabling precise drug delivery, controlled release, and reproducible therapeutic outcomes (Drais 2023; Abla et al. 2025). PDI values < 0.3 suggest high uniformity (Xiao et al. 2021); values 0.3–0.5 can be acceptable based on the application (Mostafa et al. 2015; Ibrar et al. 2022); values > 0.5 suggest broader size distributions.

Another critical parameter that predicts the stability of nanoemulsions is zeta potential, which reflects the surface charge of the droplets. Higher absolute zeta potential values (±30 mV) indicate stronger repulsion between droplets, reducing the risk of aggregation and coalescence due to electrostatic repulsion, thereby prolonging shelf life and ensuring efficient drug delivery through improved bioavailability and targeted interactions (Drais 2023; Abla et al. 2025). However, this rule can only be applied to systems stabilized by ionic surfactants. In contrast, non‐ionic surfactants generally exhibit zeta potential values closer to zero, as they do not carry any charge, yet can still provide sufficient stabilization of the formulation (Sinha et al. 2016). Therefore, although the zeta potential is a valuable indicator of stability, it is essential to consider the surfactant employed in the formulation for accurate interpretation.

Nanoemulsions have been extensively explored over the past decade for transporting pharmaceutical phytochemicals through the skin, as they enhance therapeutic efficacy, enable local or systemic action, bypass hepatic metabolism, and facilitate immediate treatment withdrawal (Morteza‐Semnani et al. 2022; Mostafa et al. 2015). Additionally, as a transdermal drug delivery system, nanoemulsions offer several advantages over other lipid‐based nanocarriers.

Compared to liposomes, nanoemulsions are more stable and effective systems for transporting lipophilic and volatile compounds, improving EOs' solubility because of their lipophilic interior (Kang et al. 2024; Chaurasiya et al. 2024). Furthermore, nanoemulsions facilitate skin penetration, a significant advantage over liposomes, which have limited deep skin penetration and often require functional surface modifications to enhance their effectiveness. Similar limitations are observed with cyclodextrin inclusion complexes, reinforcing the suitability of nanoemulsions as a cost‐effective and scalable alternative for skin delivery of EOs (Fakhraei et al. 2025; Kakadia and Conway 2022; Kang et al. 2024).

Nanoemulsions also offer a higher drug‐loading capacity, enhanced oil solubility, and longer shelf life compared to solid lipid nanoparticles (SLNs), nanostructured lipid carriers (NLCs), and polymeric nanoparticles. SLNs and NLCs, in particular, are susceptible to drug efflux from their lipid matrix caused by changes in their polycrystalline nature during storage, whereas nanoemulsions retain their content over time (Lasoń 2020; Kang et al. 2019).

Considering all these advantages and physicochemical parameters, nanoemulsions may be considered the most efficient systems for encapsulation and topical delivery of hydrophobic components, such as EOs. Overall, this nanotechnology improves EOs' stability and bioavailability, protects from volatility, enhances water solubility, and facilitates skin permeability (Mohammadifar et al. 2021; Tawfik et al. 2024).

### 4.2 Wound Healing

Healing is a natural process in which the body can compensate for damage to wounded tissue (Kazemi et al. 2020). However, this complex process involves multiple stages, requiring different cell types and signaling pathways, ultimately leading to skin regeneration (Guerrero et al. 2024). Consequently, it occurs very slowly and carries a high risk of microbial infection, reducing the quality of life and, in some cases, requiring long‐term treatments that can be expensive and complex (Bahloul et al. 2024; Kazemi et al. 2020). Current wound healing therapies often do not yield satisfactory clinical outcomes and face several challenges, including bacterial resistance and limited efficacy. Moreover, some therapies carry risks of adverse effects, such as allergic reactions, and may not be suitable for patients with specific medical conditions or compromised immune systems (Fakhraei et al. 2025).

Therefore, there is a growing interest in developing innovative strategies to enhance wound healing while minimizing the risk of infections and adverse reactions (Guerrero et al. 2024). Plants' bioactive compounds are being explored to improve these processes, and they have shown higher effectiveness and safety compared to chemical products at a more reasonable price (Bahloul et al. 2024; Kazemi et al. 2020). Recently, some EOs have been investigated for their tissue repair potential, attributed to their chemical composition, which facilitates regeneration due to their antioxidant, immunomodulatory, anti‐inflammatory, and antimicrobial properties (Mssillou et al. 2025).

For example, the incorporation of eucalyptus EO into a nanoemulsion at a concentration of 16.66% improved its bactericidal activity against the clinical pathogen *S. aureus* (Sugumar et al. 2014). The reduced droplet size (3.8 nm) increased the surface area, promoting greater interaction with bacterial membranes. Then, they evaluated the potential of the eucalyptus EO nanoemulsion in promoting tissue repair. A skin irritancy test carried out on rats (*n* = 4 per group) by application of a single dose of 100 μL of the nanoemulsion demonstrated its safety for topical application, as no signs of irritation were observed after the daily administration for three consecutive days, with formalin (0.8%) used as a positive control. Further evaluation involved applying 1 mL of nanoemulsion to the injury created in the skin of rats (*n* = 4 per group). After 16 days, complete wound closure was observed in the nanoemulsion‐treated group, while rats treated with 1 mL of neomycin ointment (positive control) showed 94.2% wound contraction.

Sweet fennel EO also has the potential for promoting tissue repair due to its antimicrobial, anti‐inflammatory, antioxidant, and analgesic properties, which help reduce swelling, oxidative stress, and pain. For this reason, a sweet fennel EO nanoemulsion was developed to enhance its therapeutic efficacy and promote regeneration (Guerrero et al. 2024). The wound‐healing potential was assessed through the topical treatment of mice‐induced skin lesions for seven consecutive days. The group treated with nanoemulsion containing 5% sweet fennel EO (*n* = 4) demonstrated a statistically significantly higher efficiency in wound closure (~100%) than the control group treated with saline solution (*n* = 4). Based on these results, the nanoemulsion was incorporated into chitosan‐PVA films since chitosan is known to accelerate healing by forming a protective barrier over the wound. At the same time, PVA offers significant properties for wound dressings, including swelling capability, water solubility, minimal toxicity, bioadhesive properties, biocompatibility, and elasticity. However, no comparison was made with pure sweet fennel EO or positive control, and the in vivo efficacy of the films was not investigated, making it not possible to attribute this effect exclusively to the applied nanotechnology.

Recently, the EO of *Pituranthos tortuosus*, a plant native to Tunisia, was explored within a nanoemulsion‐based gel (nanoemulgel) formulation as a novel delivery system for wound care (Bahloul et al. 2024). The gelling with polyacrylate crosspolymer‐6 ensured prolonged skin residence, enhancing the formulation's effectiveness. In a wound healing study on rats (*n* = 4 per group), the nanoemulgel formulated with 1% of *Pituranthos tortuosus* EO reached complete wound contraction by day 10, demonstrating significantly faster healing than the untreated group. Animals treated with a blank nanoemulsion showed suppuration and very slow progression toward healing, confirming that none of the components in this formulation, except for EO, influence the healing process. This outcome indicates significant potential for future applications in wound dressing materials, as it demonstrated the shortest wound closure time among the studies included in this review.

*Perovskia abrotanoides* Karel is a medicinal plant known to promote the synthesis of vascular endothelial growth factor (VEGF) and angiogenesis, which leads to the formation of granulation tissue and re‐epithelialization (Fakhraei et al. 2025). Therefore, the wound healing efficiency of *P. abrotanoides* EO (5%) nanoemulsion incorporated into a chitosan hydrogel (2% w/w) was evaluated (Fakhraei et al. 2025). Combining nanoemulsion and chitosan gel may improve the bioavailability, physical stability, and skin absorption of active pharmaceutical ingredients. In an antimicrobial test, the hydrogel‐thickened nanoemulsion was more effective against *P. aeruginosa* and *S. aureus* (MIC and MIB = 625 μg/mL) compared to the plain nanoemulsion (MIC and MIB > 1250 μg/mL), while the pure *P. abrotanoides* EO exhibited superior activity against *S. aureus* (MIC = 312.5 μg/mL; MIB = 625 μg/mL) and *C. albicans* (MIC = 156.52 μg/mL; MIB = 312.5 μg/mL). In the wound healing test on rats (approximately 0.5 mL of formulation per rat), the hydrogel‐thickened nanoemulsion achieved complete wound contraction by day 21, comparable to the standard group (phenytoin cream), and reduced inflammation levels compared to the untreated group, highlighting its potential to prevent infection and accelerate skin regeneration. Nevertheless, the exact number of animals used in each group was not specified, representing a significant limitation.

Lavender EO is already widely used to improve healing processes, primarily due to its main components, linalool and linalyl acetate. The incorporation of 2% of lavender EO into a nanoemulsion cream, combined with 2% of licorice ( *Glycyrrhiza glabra* ) extract, was investigated for its potential in deep skin wound healing and antioxidant activity (Kazemi et al. 2020). In a study on rats (*n* = 17 per group), the nanoemulsion‐cream and phenytoin 1% ointment (positive control) groups showed the most uniform collagen fibers, faster re‐epithelialization, and higher fiber density and thickness. By day 14, a complete wound closure occurred only in these two groups, outperforming the untreated and placebo groups. Additionally, the nanoemulsion increased the expression of TGF‐β1, type I, and type III collagen genes, as well as antioxidant enzyme activity in rat skin tissues, suggesting the potential of a nanoemulsion formulation containing lavender EO and licorice extract to improve wound healing.

Similarly, lavender EO (1%) nanoemulsion was formulated with biosafety and promising effectiveness in burn wound healing (Miastkowska et al. 2023). Through a scratch assay with HaCaT cells, the nanoemulsion promoted cell migration, resulting in approximately 50% induction of scratch closure within 24 h, whereas pure lavender EO had no effect, likely due to its limited solubility in the culture medium. The nanoemulsion also influenced cytokine stimulation, upregulating VEGF‐A and IL‐8 genes, and showed no hemolytic activity, highlighting its potential biocompatibility for topical applications.

However, the main components of lavender EO, considered crucial for skin regeneration, can cause irritation (erythema or edema) and allergy upon direct contact with the skin. These reactions can be mitigated by using nanoemulsion formulations. The nanoemulsion application caused no skin irritation in rabbits (*n* = 3) after 24 h of occlusive exposure using a patch. However, in skin sensitization studies in pigs (*n* = 18) conducted under the same conditions, higher concentrations (above 70% nanoemulsion in an occlusive patch) induced moderate sensitization, suggesting deeper penetration and stimulation of dermal cells. This indicates the importance of optimizing the EO concentration in nanoemulsions to regulate penetration depth and active compound levels in skin layers.

**FIGURE 4:**
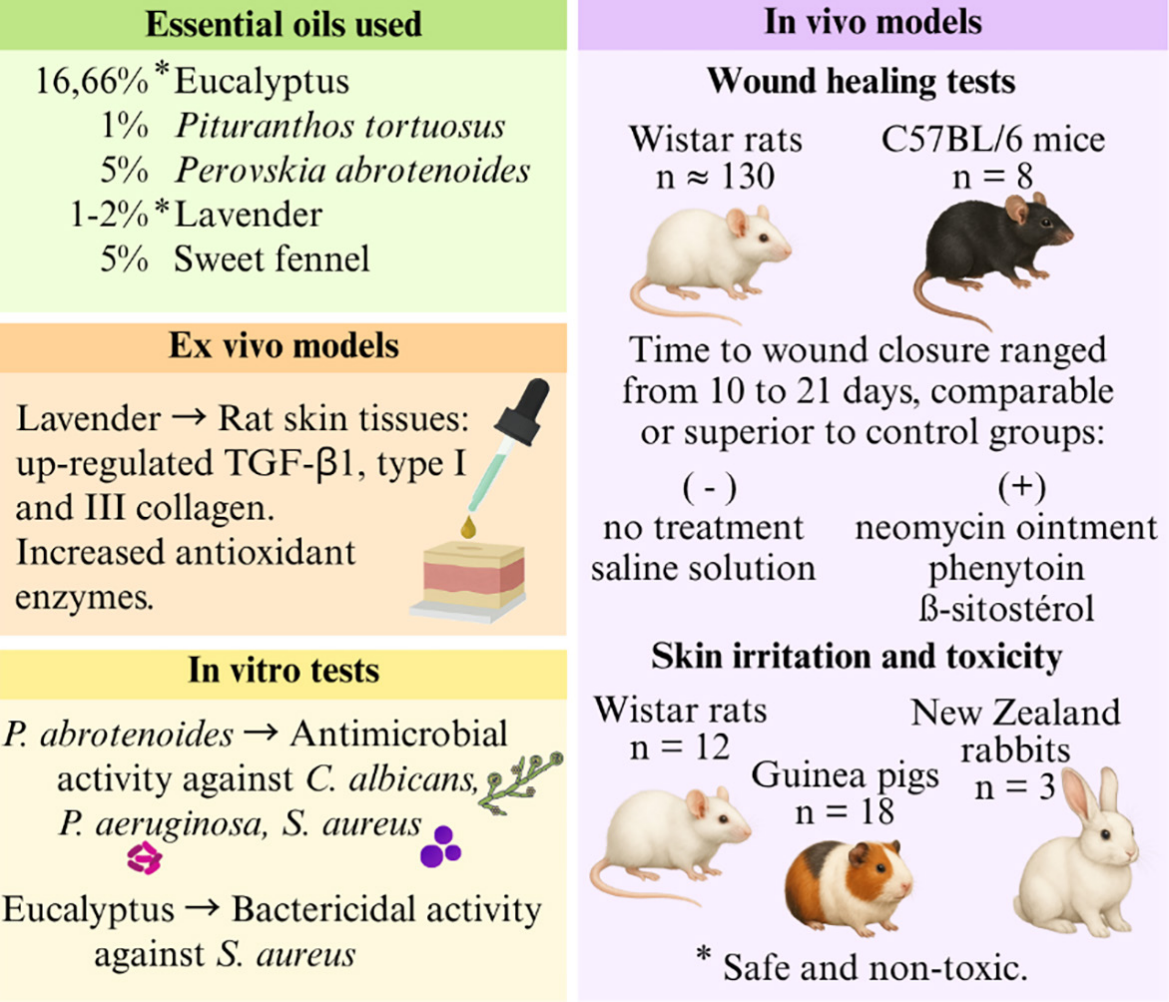
Summary of the potential activity of EOs incorporated into nanoemulsions for wound healing applications.

All studies included in this review that investigated the wound healing effects of EOs reported enhanced activity when incorporated into nanoemulsions, outperforming other treatments such as pure EO, negative controls, and conventional formulations. In some cases, the results were comparable to or even superior to those of the positive control groups. The nanoemulsion containing *Pituranthos tortuosus* (Coss.) Maire EO promoted faster wound closure, achieving complete healing within 10 days (Figure 4).

However, variations between in vivo assays across studies make it difficult to determine conclusively whether this EO is the most effective for wound healing. Moreover, no consistent pattern was identified regarding the physicochemical characteristics of the nanoemulsions, nor was there a clear relationship between the nanoemulsification techniques employed and the outcomes.

### 4.3 Anti‐Inflammatory and Analgesic

Inflammation is a crucial component of the immune system's initial response to infection or injury, aimed at eliminating the initial cause and preserving cellular and tissue structure, ultimately maintaining homeostasis. However, persistent and uncontrolled inflammatory responses can cause severe tissue damage (Sousa et al. 2023; Xiao et al. 2021). The inflammation process involves various events, including an increase in vascular permeability, provoking an exudation of fluids from blood into the interstitial space; infiltration of leucocytes from blood into the tissue; activation of mitogen‐activated protein kinases (MAPK), which stimulates the release of pro‐inflammatory cytokines and mediators such as TNF‐α and interleukins; and granuloma formation and tissue repair (Kreutz et al. 2023). Inhibiting the excessive production of IL‐1β, IL‐6, IL‐8, and TNF‐α, along with inflammatory mediators such as nitric oxide (NO) and free radicals, as well as Prostaglandin E2 (PGE2), can prevent or mitigate many inflammatory diseases (Xiao et al. 2021; Zhao et al. 2023).

Atopic dermatitis is one of the most prevalent chronic inflammatory skin diseases, characterized by dry, eczematous lesions with intense pruritus. Chamomile EO, derived from *Matricaria chamomilla* , possesses a range of beneficial properties that have demonstrated efficacy in treating atopic dermatitis. However, its application is limited by low solubility and instability, primarily due to poor compatibility with substrates (Xu et al. 2024).

To overcome these limitations, a chamomile EO nanoemulsion thickened with *Bletilla striata* polysaccharide gel was developed to enhance its stability and moisturizing properties, accelerating skin repair (Xu et al. 2024). The efficacy of the nanoemulgel, evaluated on an atopic dermatitis‐like mouse model induced by 2,4 dinitrofluorobenzene (DNFB) (*n* = 5 per group), demonstrated a significant reduction in epidermal thickness, mast cell infiltration, and cytokine expressions (IgE, TNF‐α, and IL‐4) in both serum and skin tissues, showing superior efficacy compared to pure chamomile EO and comparable to the standard group (dexamethasone). Additionally, the formulation was safe, as no severe damage to hepatic and renal functions was observed in blood analyses from treated animals (*n* = 5). These results support its potential use in modulating the immune response and mitigating inflammation associated with atopic dermatitis. However, as a chronic disease, atopic dermatitis often requires prolonged treatment, and the effects of continuous use of this nanoemulgel over time remain unexplored.

Psoriasis is another chronic and recurrent inflammatory skin disease characterized by erythematous plaques with severe itching and silvery‐white surface scales, commonly affecting the elbows, knees, buttocks, palms, and scalp. These symptoms result from epidermal hyperproliferation, keratinocyte dedifferentiation, increased angiogenesis in the subepidermal layer, and inflammatory cell infiltration, which stimulate the release of pro‐inflammatory mediators. Herbal medicine has been gaining interest as an alternative treatment, as its long‐term use may lead to immunosuppression, increased susceptibility to infections, and delayed wound healing (Tawfik et al. 2024).

Thus, the anti‐psoriatic effect of *Artemisia monosperma* EO (1%) nanoemulsion was evaluated using the imiquimod‐induced psoriatic model in mice (*n* = 6 per group) (Tawfik et al. 2024). The nanoemulsion decreased psoriasis area and severity index (PASI), downregulated the level of NF‐κB (currently elevated in psoriasis), and attenuated the expression of inflammatory mediators TNF‐α, IL‐6, and IL‐17, with a nonsignificant difference from the standard group (tacrolimus cream). The anti‐psoriatic effect was attributed to the main components of *Artemisia monosperma* EO, acenaphthene (an aromatic hydrocarbon), and sabinene (a monoterpene), due to their high skin penetration ability and absorption. Nevertheless, a group treated with pure *Artemisia monosperma* EO was not included due to the volatilization of the EO and the inability to achieve sufficient skin‐contact time, limiting the capacity to affirm the benefit of its incorporation into a nanoemulsion.

Incorporating EOs into nanoemulsions has proven to be an effective strategy to enhance their bioavailability, permeation, and controlled release. However, some skin conditions require thicker formulations. Thus, hydrogel‐thickened nanoemulsions are obtained by adding polymers that increase viscosity, improve skin adhesion, provide a more pleasant sensory experience, and prolong residence time in the skin, thereby reducing the dose and frequency of application (Kreutz et al. 2021; Carneiro et al. 2022).

Hydroxyethylcellulose and chitosan hydrogel‐thickened nanoemulsions containing *Aniba canelilla* (Kunth) Mez EO (10%) were produced for the treatment of cutaneous inflammatory processes and were more effective in controlling and sustaining the EO in vitro release through synthetic mixed cellulose ester membranes than pure *Aniba canelilla* (Kunth) Mez EO and plain nanoemulsion (Kreutz et al. 2023). Ex vivo permeation and retention studies in porcine skin showed higher epidermal and dermal retention for plain nanoemulsions and hydrogels than pure *Aniba canelilla* (Kunth) Mez EO, highlighting their potential to act on local inflammatory processes. However, chitosan hydrogel‐thickened nanoemulsion showed low stability and was rejected. The anti‐inflammatory activity, evaluated using a Croton oil‐induced mouse ear edema model (*n* = 6–7 per group), demonstrated similar reductions in ear weight, neutrophil infiltration, and IL‐1β levels between the pure *Aniba canelilla* (Kunth) Mez EO, plain nanoemulsion, and hydroxyethylcellulose hydrogel‐thickened nanoemulsion. These results suggest that the therapeutic effect is primarily attributed to the *Aniba canelilla* (Kunth) Mez EO, and that hydroxyethylcellulose, used as a thickener, did not affect efficacy. Future research should focus on evaluating the safety of this formulation by investigating potential skin reactions.

One of the primary and most debilitating symptoms of inflammation is pain. Several researchers have revealed the analgesic properties of the main constituents of EOs, and some of them present a high potential as alternatives to nonsteroidal anti‐inflammatory drugs (Aziz et al. 2019).

Therefore, the effect of nanoemulsion containing rosemary and peppermint EOs, known for their anti‐inflammatory, antioxidant, and antinociceptive effects, was explored to reduce osteoarthritis pain (Mohammadifar et al. 2021). Osteoarthritis is a musculoskeletal disorder characterized by the progressive destruction of articular cartilage, local inflammation, and joint pain. The treatment is symptomatic with analgesic and anti‐inflammatory drugs (Mohammadifar et al. 2021). Firstly, in an acute irritation study conducted on rabbits (*n* = 3) and an acute dermal toxicity study involving female (*n* = 3) and male (*n* = 3) rabbits, no severe skin reaction was observed after 4 h of exposure to nanoemulsion, and no abnormality was noted in the skin, eyes, mucous membranes, and behavioral patterns 14 days post‐exposure. Then, the nanoemulsion antinociceptive effects were evaluated in a rat model of osteoarthritis induced by intra‐articular injection of monosodium iodoacetate (*n* = 36, 6 per group). Behavioral tests were conducted by measuring the paw withdrawal response to various stimuli after the application of nanoemulsions. Nanoemulsion significantly decreased mechanical allodynia, cold allodynia, and thermal hyperalgesia more than pure rosemary and peppermint EOs, and it was comparable to the positive control (1% diclofenac sodium). The ambulatory‐evoked pain score and oxidative stress significantly decreased with all treatments compared to the control group (no treatment). Nevertheless, the amount of rosemary and peppermint EOs in the formulations was not specified, making it difficult to assess their contribution to the observed effects accurately.

Eucalyptus EO is also widely discussed for its natural analgesic properties, which have demonstrated effectiveness in treating muscle aches. The transdermal analgesic potential of eucalyptus EO (2.5%) nanoemulsion was assessed through an in vivo hot plate test in rats (*n* = 6 per group) (Aziz et al. 2019). Nanoemulsion was topically applied to the limbs of rats (100 mg/kg), which were then placed on a hot plate at a constant temperature of 55°C. The nanoemulsion administration significantly prolonged the rats' response to thermal pain, compared to the negative control (treated with normal saline, 10 mL/kg), and was slightly better than pure eucalyptus EO (500 mg/kg). The successful nanoemulsion analgesic potential suggests its inhibitory effects on both central and peripheral analgesic activities. Since only a single concentration was tested for each group, and the administered doses differed between the nanoemulsion and pure eucalyptus EO groups, further studies should investigate a potential dose‐dependent effect. This would help establish the effective dose and therapeutic window for potential clinical applications.

**FIGURE 5:**
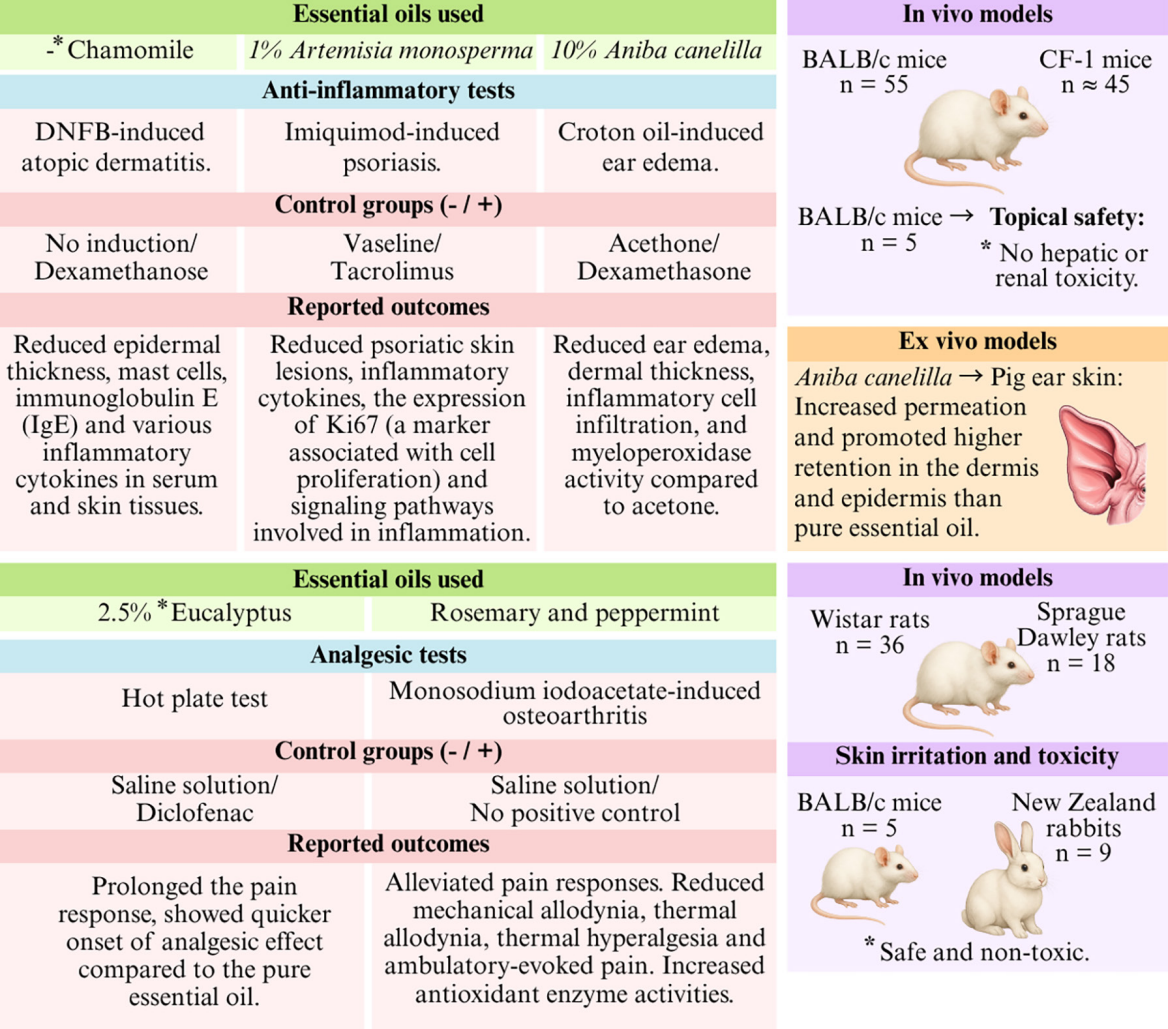
Summary of anti‐inflammatory and analgesic activity of EOs incorporated into nanoemulsions.

Despite these promising findings, the diversity of animal models, experimental designs, and the outcome measures assessed, ranging from behavioral pain responses to biochemical inflammatory markers and histological changes, limits direct comparison across studies (Figure 5). Additionally, the chemical composition of the EOs and physicochemical characteristics of nanoemulsions were often underreported or varied considerably, limiting insight into how these factors influence bioactivity or skin penetration.

### 4.4 Cosmetics

EOs as active ingredients in pharmaceutical products are gaining increasing attention due to their broad range of bioactivities and their Generally Recognized as Safe (GRAS) status (Shakeel et al. 2021). A similar trend is observed in the cosmetics market, driven by growing consumer preference for sustainable and natural products. However, incorporating plant‐based actives into functional cosmetics presents technical challenges, including limited solubility, stability, skin permeability, and potential skin irritation (Nhani et al. 2024). Thus, nanoemulsions present a promising approach to enhancing cosmetic performance and skin properties, since smaller particles enhance texture, facilitate absorption, increase effectiveness, and ensure greater stability (Pandey et al. 2024).

The incorporation of lavender EO into nanoemulsions provided distinct advantages over conventional emulsions, particularly in hydration and skin barrier function (Flekka et al. 2024). The effects of the topical application of lavender EO emulsion and nanoemulsion on the skin were tested in vivo on 10 healthy volunteers using non‐invasive techniques. Each formulation was randomly applied to either the left or right forearm, with the untreated side serving as a control. Thirty minutes later, both formulations showed a significant increase in skin hydration compared to baseline. From 1 h onwards, nanoemulsion demonstrated greater skin hydration enhancement than conventional emulsion. Then, a cotton bud impregnated with acetone induced barrier disruption on untreated skin. Notably, only the nanoemulsion reduced transepidermal water loss (TEWL) compared to untreated skin, indicating full epidermal barrier recovery within 30 min. These findings underscore the potential of nanoemulsion as an effective lavender EO carrier in skincare formulations, being a promising candidate for scalability.

Acne vulgaris is a common inflammatory disorder of the pilosebaceous unit, caused by various factors, including abnormal keratinization resulting in comedones, altered sebum production regulated by androgens, and the presence of *Cutibacterium acnes* (previously called *Propionibacterium acnes* ) and *Staphylococcus epidermidis* , which cause skin irritation (Shakeel et al. 2021; Infante, Campos, et al. 2023). Given its multifactorial nature, cosmetic formulations containing bioactive compounds with antimicrobial and anti‐inflammatory properties can be effective anti‐acne products.

The most extensively studied EO for acne treatment is tea tree oil extracted from *Melaleuca alternifolia*. Therefore, a randomized, placebo‐controlled, double‐blind clinical in vivo study evaluated the efficacy of a cosmetic formulation for non‐inflammatory acne using tea tree EO nanoemulsion at a concentration of 2% (Infante, Darvin, and Campos 2023). A total of 53 male participants aged 18–28 years with non‐inflammatory acne were randomly divided into four groups: nanoemulsion, the vehicle with 2% of pure tea tree EO, a mixture of four EOs (tea tree, lavender, eucalyptus, and tangerine (1:1:1:1), total 2%), and a placebo. All participants used sunscreen with SPF 50 daily for 15 days. Then, they topically applied their assigned formulation to the facial region, 1 mL per day before bedtime, while using sunscreen. After 90 days of treatment, the nanoemulsion group demonstrated the most promising outcomes in reducing the number of comedones and the area of pilosebaceous units, indicating a decrease in sebum and protein accumulation. However, all groups showed some improvement in non‐inflammatory acne, which could be attributed to the regular application of sunscreen, as unprotected sun exposure can exacerbate follicular hyperkeratinization.

Tea tree EO has also shown potential in combating skin aging due to its high antioxidant capacity (Infante, Campos, et al. 2023). Skin aging is a gradual and complex process characterized by various adverse esthetic manifestations, including pigmentation disturbances, wrinkles, dehydration, and roughness (Infante, Campos, et al. 2023; Ranjbar et al. 2025). Chronic exposure to solar radiation accelerates aging by generating reactive oxygen species in the skin, resulting in chronic oxidative stress. This process, known as photoaging, damages proteins, lipids, and DNA, and activates enzymes such as collagenase and elastase, which degrade collagen and elastin—the key molecules responsible for maintaining skin integrity (Ranjbar et al. 2025). Thus, incorporating antioxidant compounds into topical formulations is crucial for preventing or reducing photoaged skin (Infante, Campos, et al. 2023).

In another study by Infante et al., a tea tree EO nanoemulsion was demonstrated to be a promising cosmetic product for improving photoaged skin when used in combination with sunscreen (Infante, Campos, et al. 2023). An ex vivo study using porcine ear skin showed that the nanoemulsion enhanced tea tree EO penetration compared to its pure form. While 90% of the pure EO remained in the *stratum corneum*, approximately 26% of the nanoemulsion reached the viable epidermis, thereby improving the anti‐aging effect of the active compound. A clinical study was carried out with 40 male participants aged 18–28 years, randomly divided into three groups: nanoemulsion (2% in a vehicle), pure tea tree EO (2% in a vehicle), and placebo. The same treatment protocol described above was followed (Infante, Darvin, and Campos 2023). After 90 days of treatment, the nanoemulsion improved hydration in deeper skin layers, reduced *stratum corneum* thickness, increased keratinocyte area in the *stratum granulosum*, and enhanced skin morphology and structure, even with a reduced quantity of EO than the pure tea tree EO group. Nevertheless, both groups reduced the skin surface sebum and increased the collagen fiber density in the papillary dermis, likely due to the chemical composition of tea tree oil.

Nanoemulgels also offer several advantages for cosmetic applications due to their higher viscosity, jelly‐like consistency, improved applicability, thixotropic behavior, greaseless nature, enhanced spreadability, and controlled rheological properties (Sinha et al. 2016). Therefore, a tea tree EO nanoemulgel exhibited a higher permeation rate for topical release than a conventional gel, both of which were formulated with 5% tea tree EO concentration. Additionally, the therapeutic efficacy against bacterial strains ( *S. aureus* , *S. mutans* , *E. coli* , and *P. aeruginosa* ) and fungal strains ( *Candida albicans* ) was significantly improved compared to the conventional gel and pure tea tree EO. Primary skin irritation studies on rabbits (*n* = 6) ensured that the nanoemulsion exhibited barely perceptible skin irritation 72 h after application, indicating it was considered non‐irritant at the applied dose (500 mg), highlighting its suitability for topical application.

The potential of EOs in improving photoaged skin is aligned with the findings from Ranjbar et al., who demonstrated that a nanoemulgel containing *Rosa damascena* EO mitigated UVB‐induced dermal damage and wrinkle formation in rats (Ranjbar et al. 2025). The DPPH assay revealed that the nanoemulgel exhibited significant antioxidant effects, higher than pure *Rosa damascena* EO. The inhibitory effect of collagenase and elastase was also substantially higher for the nanoemulgel. An in vivo study using a rat model of UVB‐induced extrinsic aging showed that the nanoemulgel significantly reduced the development of coarse wrinkles on the foot skin compared to the control (no treatment) and pure *Rosa damascena* EO groups. However, they tested a preventive treatment by applying the products to the rats' foot skin before UVB exposure, and no significant photoprotective activity was observed in any of the groups, as evidenced by visible signs of photoaging (wrinkle formation), suggesting that a post‐exposure treatment is more effective.

Additionally, the antibacterial activity against *P. aeruginosa* , *S. aureus* , and *E. coli* was evaluated, with the best efficacy observed against *P. aeruginosa* . Although the results are promising, their interpretation and generalization are limited by the small sample size used in the study (approximately 4–5 rats per group), which reduces the statistical power and robustness of the findings. Consequently, larger sample sizes are required to validate and strengthen the evidence.

The EO antibacterial activity was explored by Drais, who developed a nanoemulgel skin sanitizer using peppermint and myrtle EOs as an alternative to alcoholic products (Drais 2023). Alcohol‐based hand gel is an antiseptic product used to remove common pathogens when soap and water are unavailable. However, alcohol can cause skin dryness, destroy lipid barriers, and eventually cause hand eczema and dermatitis, associated with symptoms like acne, wrinkles, burning, swelling, erythema, and cracking. The nanoemulgel developed was tested on 30 volunteers, and no signs of irritation were found, indicating the formulation was safe for topical application. The antimicrobial activity against *E. coli* and *S. aureus* increased with higher concentrations of peppermint and myrtle EOs. All formulations were more effective against *E. coli* , and *S. aureus* was relatively more sensitive to formulations containing myrtle oil than those containing peppermint oil at the same concentrations. Moreover, the mixture of both peppermint and myrtle EOs enhanced the antimicrobial activity, making it a promising product for sanitizing human skin.

**FIGURE 6:**
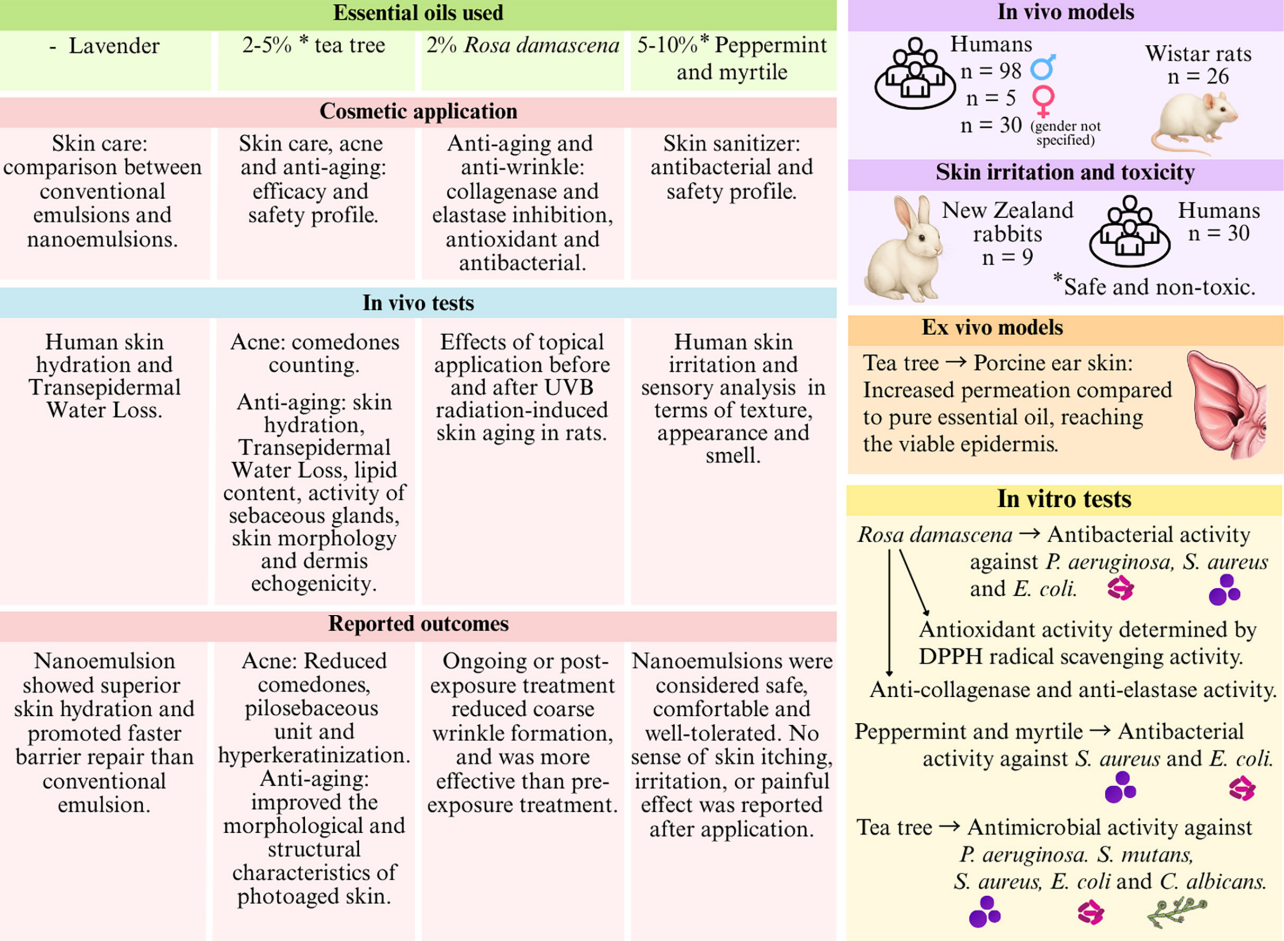
Summary of potential cosmetic applications of EOs incorporated into nanoemulsions.

The reviewed studies highlight the growing interest in utilizing EO nanoemulsions for cosmetic purposes, as they enhance the efficacy and tolerability of EOs in skincare. Tea tree EO was the most studied due to its well‐established cosmetic properties. Notably, when incorporated into a nanoemulsion, tea tree EO penetrated more deeply into the skin, demonstrating improved skin hydration in both clinical and preclinical models, reduced sebaceous secretion, increased collagen fiber density, and a significant reduction in non‐inflammatory acne lesions. These findings underscore the potential of nanoemulsions to enhance bioavailability and dermal delivery of EOs (Figure 6).

Across all studies, EOs nanoemulsions demonstrated good skin compatibility, with no significant irritation or adverse effects reported in either animal models or human participants. This is critical for cosmetic formulations, where long‐term use and sensory properties significantly impact product acceptance. Nevertheless, several limitations prevent robust comparisons between studies, including variations in formulation excipients, viscosity (nanoemulsion vs. nanoemulgel), dosage regimens, sample sizes, treatment durations, and the use of additional products (e.g., sunscreen).

### 4.5 Transdermal Delivery and Permeation Enhancer

The skin is one of the most accessible organs in the human body, making it an ideal target for drug delivery, which is classified as dermal when the target is the skin itself or transdermal when the drug must pass through the skin layers to reach its target (Lasoń 2020). However, a small number of formulations can permeate the skin sufficiently to exert a therapeutic effect due to the *stratum corneum* barrier function (Morteza‐Semnani et al. 2022). One approach to overcoming this barrier is to utilize permeation enhancers in the formulation to enhance drug penetration into the deeper layers of the skin and potentially achieve systemic effects (Lasoń 2020; Morteza‐Semnani et al. 2022).

Terpenes and terpenoids have attracted significant interest as permeation enhancers due to their natural origin and generally low toxicity and irritancy potential compared to other synthetic materials. Additionally, the EOs' therapeutic potential for topical administration can yield a synergistic effect, prolonging efficacy and improving overall treatment outcomes (Lasoń 2020).

**FIGURE 7:**
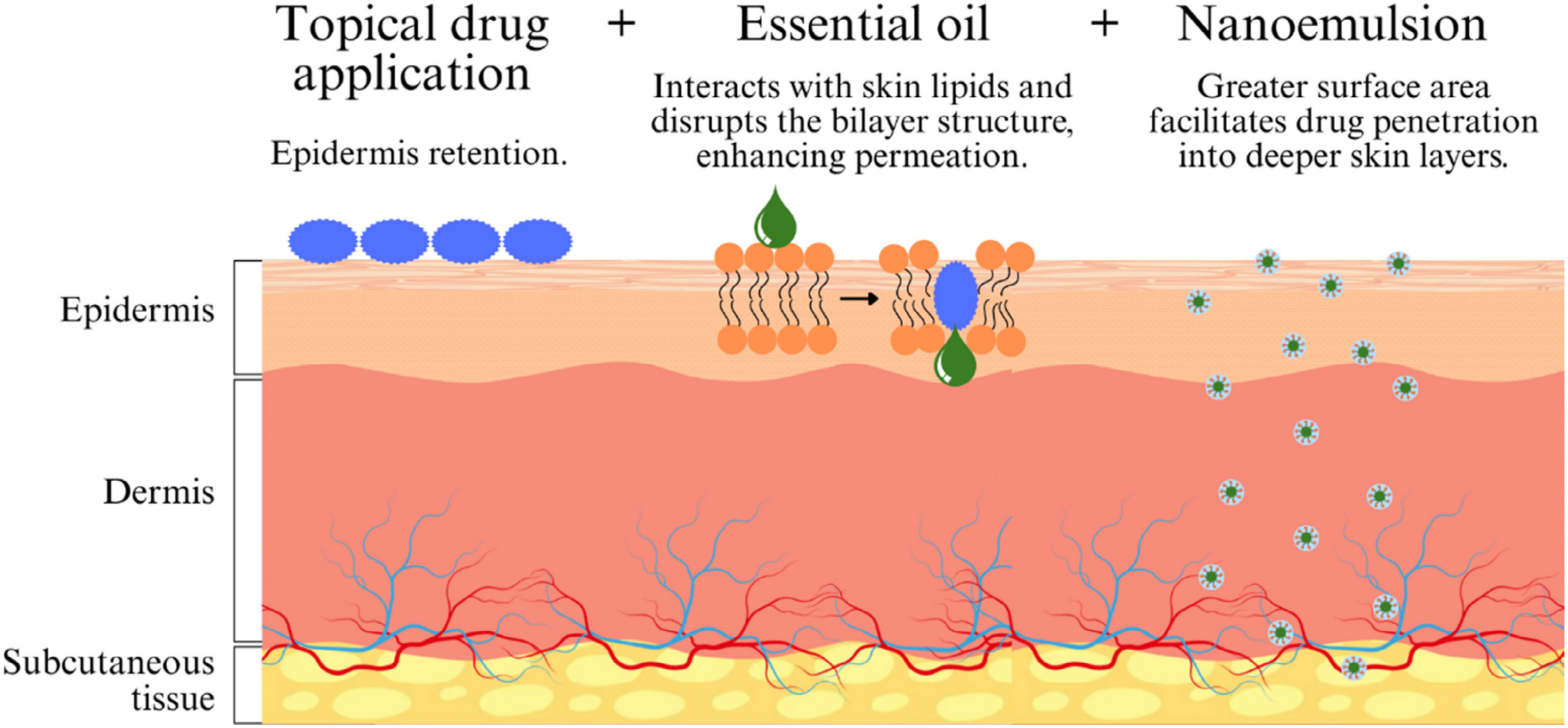
Mechanisms of EOs as permeation enhancers and nanoemulsions in facilitating skin penetration.

Skin absorption and permeability of active substances can be further enhanced by incorporation into a nanostructured system, as the nanometric droplet size provides a greater surface area, making these systems suitable for transdermal drug delivery (Figure 7) (Mohammadifar et al. 2021; Kakadia and Conway 2022). The oil phase facilitates interaction with the lipid layers of the stratum corneum, disrupting the bilayer structure and compromising its barrier function, while the water phase increases the interlamellar volume, creating voids that enhance drug permeability through the lipid pathway (da Silva et al. 2019).

Cumin EO was used to enhance the skin absorption of diclofenac sodium, a potent nonsteroidal anti‐inflammatory drug, through the incorporation of the drug into a nanoemulgel containing cumin EO (Morteza‐Semnani et al. 2022). An in vitro permeation study using rat abdominal skin revealed that formulations with higher concentrations of cumin EO significantly increased diclofenac permeation and enhanced the epidermal/dermal levels of the drug at 24 h by almost 18 times more than a simple gel, indicating the prolonged effect. The analgesic effects were established through a tail flick test in mice (*n* = 6), which measured the latency time to respond to nociceptive pain stimuli, and a formalin‐induced inflammatory pain test in mice (*n* = 6), which assessed the time spent licking the paws after formalin injection. The cumin EO enhanced the analgesic activity of diclofenac compared to the blank group, simple gel, and marketed formulation.

Geranium EO was also explored for its antimicrobial and permeation‐enhancing activity to develop nanoemulsions loaded with pravastatin for the transdermal management of burn wounds (Rizg et al. 2022). Pravastatin is a statin drug with desirable effects on wound healing due to its ability to induce angiogenesis. Therefore, burn wounds were induced on rat skin (*n* = 69, divided into 23 groups), and each group was treated once daily for 14 days with one of the nanoemulsions developed by the experimental design, containing different concentrations of pravastatin and geranium EO. Among all formulations, the optimum nanoemulsion (275 mg of geranium EO and 40 mg of pravastatin, as determined by a Box–Behnken design) resulted in the lowest mean burn wound diameter and IL‐6 serum level after 14 days, indicating a synergistic effect between geranium EO and pravastatin. These results were corroborated by an ex vivo skin permeation study using rat abdominal skin, in which the optimal nanoemulsion demonstrated a 7.6‐fold enhancement in pravastatin permeation compared to the pravastatin aqueous dispersion, and 2.7‐fold compared to the simple pravastatin/geranium EO mixture. The optimal nanoemulsion also increased antimicrobial activity against *S. aureus* by 4‐fold compared to formulations without EO, which is likely attributed to the interaction between the nanosized droplets and the geranium EO terpenes with bacterial cell membranes, causing leakage of cellular components.

Various skin conditions can also affect cutaneous appendages, such as hair, nails, and glands. Androgenetic alopecia is a disorder that targets hair follicles explicitly and is one of the most common causes of progressive hair loss, yet only a limited number of medications are approved for its treatment. In this context, metformin has emerged as a promising candidate for treating alopecia; however, its limited penetration may reduce its therapeutic efficacy. Additionally, rosemary EO contains rosmarinic acid, carnosol, and caffeic acid, which can boost microcapillary perfusion, along with 2‐methoxy carnosic acid, a diterpene with the ability to stimulate hair growth (Abla et al. 2025). Thus, metformin was loaded into rosemary EO nanoemulgel to upgrade anti‐hair loss activity. In a dialysis bag study, the nanoemulgel controlled metformin release over 24 h, likely due to its high solubilization within the nanoemulsion. The hair growth study in shaved rats (*n* = 10 per group) treated topically daily for 35 days revealed a higher number of hair follicles in animals treated with metformin loaded into a rosemary EO nanoemulgel than in other groups (untreated animals, metformin‐loaded gel, and unloaded rosemary EO nanoemulgel), with results comparable to minoxidil (positive control). Follicles were distributed throughout the epidermis, dermis, and deeper layers, emphasizing the role of rosemary EO as a penetration enhancer, its synergistic pharmacological effects with metformin, and the efficiency of the prepared formulation in promoting hair growth. The study involved a sufficiently large and well‐distributed number of animals in each group, providing reliable results. Additional clinical trials may support the potential scalability of the product for commercial use.

The antimicrobial potential of EOs has been widely explored as a good and safe alternative to current therapeutic drugs since microorganisms have developed multidrug resistance over time, requiring more efficient approaches (Ibrar et al. 2022). In this context, nine nanoemulsion formulations composed of 0.1% garlic and ginger EOs combined with different concentrations of neomycin sulfate (0.001%–0.1%) improved efficacy in treating infections and skin wounds (Ibrar et al. 2022). In general, nanoemulsions containing only garlic EO exhibited higher antimicrobial activity against *Bacillus spizizenii*, *E. coli* , and *Salmonella enterica* , while ginger EO was more effective against *S. aureus* . Interestingly, the mixture of both EOs did not affect each other's activity, likely due to their antagonistic effect in a cumulative setup. Neomycin ointment showed no activity against any of the tested microbes, confirming their resistance to the drug and emphasizing the garlic and ginger EOs' potential to enhance its effectiveness. In the wound healing activity, evaluated using an excision wound model in rabbits (*n* = 3 per group), all nine nanoemulsions significantly enhanced wound contraction, compared to neomycin sulfate ointment, resulting in complete healing within just 9 days. However, the limited sample size may not accurately represent the broader population's response to the treatment.

Dermatophytosis is a fungal infection that affects the skin of millions of people worldwide, and the primary medical treatment consists of topical antifungal therapy. However, systemic antifungals, such as ketoconazole, have been associated with severe adverse events and the development of antimicrobial resistance (Ahmad et al. 2023). The combination of ketoconazole with eucalyptus (15%) and clove (20%) EOs in a nanoemulgel was a strategy explored to enhance antifungal activity and drug permeability, enabling the reduction of the ketoconazole dose (Ahmad et al. 2023). Antifungal activity was studied in the *C. albicans* strain, and it was found that the developed formulations more effectively prevented fungal growth compared to a marketed ketoconazole formulation. An ex vivo test using rat skin showed that nanoemulgels containing eucalyptus and clove EOs enhanced ketoconazole permeation and retention and increased the spreadability, stability, and skin hydration compared to a pure ketoconazole aqueous suspension, while exhibiting a controlled release pattern. Additionally, all formulations were considered safe for topical application, demonstrating excellent tolerance in the in vivo rat skin model (*n* = 6 per group) compared to the positive control (formalin 0.7%).

**FIGURE 8:**
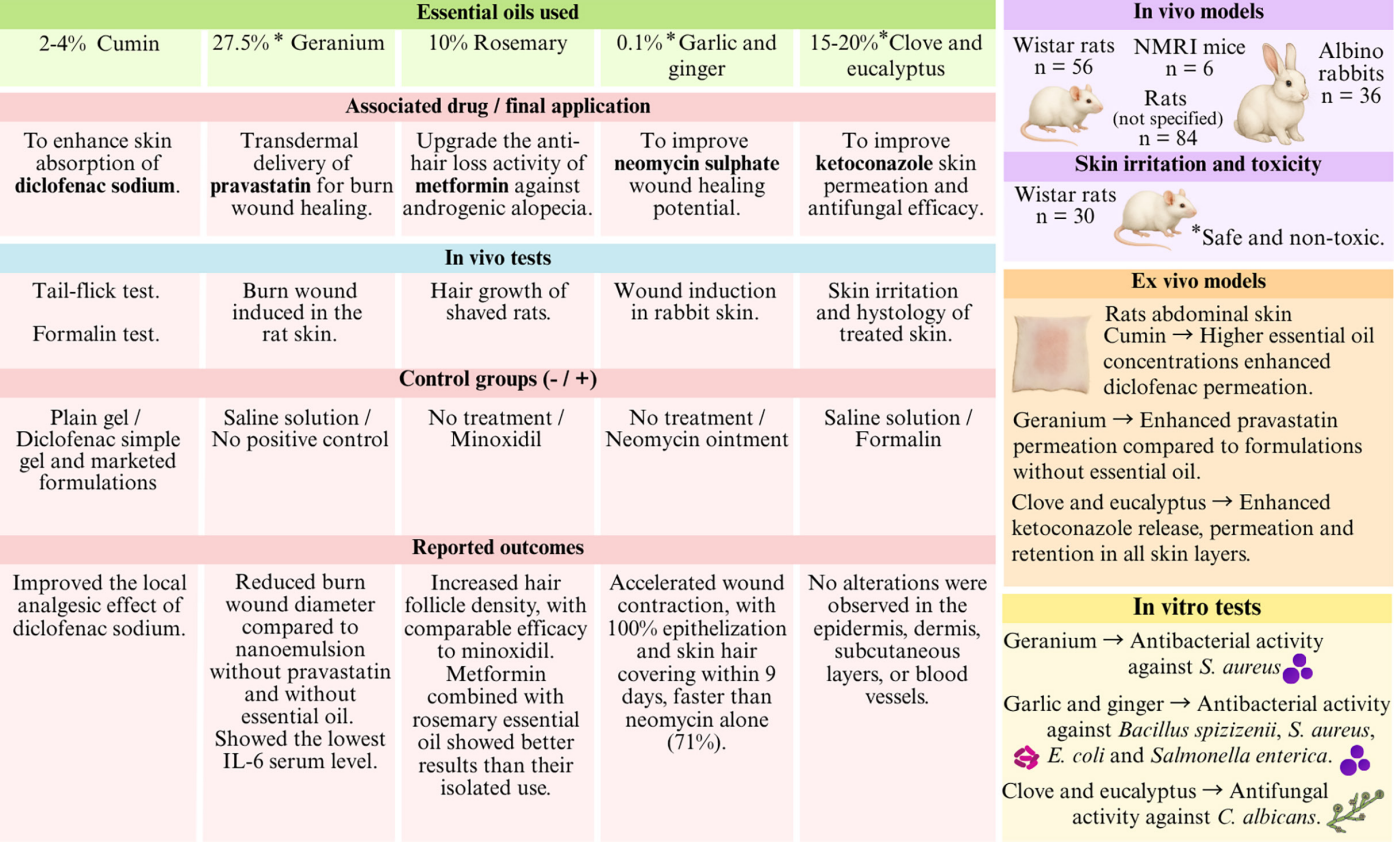
Summary of EOs incorporated into nanoemulsions as penetration enhancers for transdermal application of other drugs.

The EOs' ability to enhance the permeation of other drugs was highlighted by the reviewed studies, particularly when incorporated into nanoemulsions, which improves drug delivery and therapeutic outcomes across various topical applications. However, as observed in other sections of this review, considerable variability exists in experimental designs, EO concentrations, chemical composition, formulation characteristics, and the presence of co‐administered drugs, which challenges the direct evaluation of permeation enhancement efficacy. Moreover, the lack of standardized assessment protocols for permeation or bioavailability restricts the ability to determine which EO or formulation strategy is most effective (Figure 8).

## 5 Strengths and Limitations

To ensure a comprehensive analysis, a precise literature search was conducted across three databases without restrictions, allowing a broad overview of the topic. The PRISMA checklist was followed to ensure methodological rigor and transparency in reporting. Including only in vivo studies allowed for a more clinically relevant discussion of the effectiveness and potential of nanoemulsions in improving EOs' activity in skin conditions. Furthermore, data were analyzed independently and in a blinded manner by two reviewers to ensure reliability and quality.

A strength of this review is the systematic comparison of nanoemulsion formulations containing different EOs for skin applications. Nevertheless, many studies did not report detailed compositional analyses or quantify major active compounds of the EOs. There is a lack of consistent data correlating the chemical composition of EOs with therapeutic outcomes, which limits a reliable comparison between them.

The reporting and risk of bias assessments indicated that many studies present low methodological quality, particularly in terms of randomization and blinding. The heterogeneity of study designs, formulation characteristics, and outcome evaluation methods makes correlation and comparison of findings difficult. Therefore, conducting a meta‐analysis was not feasible. These limitations should be considered when interpreting the conclusions from the included studies, as they may affect the reliability of the results by potentially overestimating or underestimating the observed effects.

However, this issue is commonly observed in other systematic reviews involving animal studies. Therefore, there is an urgent need for improvements in animal research, especially in the dermatological field, where animal models are widely used. It is also necessary to conduct more clinical trials to translate the findings from animal studies to human applications, ensuring the efficacy and safety of EOs nanoemulsion formulations for dermatological use.

## 6 Conclusion and Future Perspectives

The incorporation of natural products into topical formulations has gained significant interest, driven by consumer demand for safer, more sustainable, and cost‐effective alternatives. This growing trend underscores the relevance of nanoemulsion as a delivery system to enhance the efficacy and applicability of EOs in dermatological products.

This review demonstrates progress in evaluating EOs for topical application, with 18 species showing enhanced efficacy when incorporated in nanoemulsions. The most frequently reported advantages of EOs nanoemulsions include the reduction in particle size, which leads to an increased surface area, along with higher stability and improved solubility of EOs.

EOs' nanoemulsions have shown promising results in preclinical studies. However, their translation to clinical or commercial applications presents significant challenges. Key issues include scalability of production, long‐term physicochemical stability, regulatory approval, and manufacturing costs. Based on the current literature, it is not yet possible to determine which EOs or nanoemulsion compositions hold the most significant promise for real‐world application. The diversity of study designs, formulation variables, and tested endpoints limits direct comparison and generalization of results. Additionally, the specific regulatory pathway may vary depending on whether the nanoemulsion is intended for cosmetic, pharmaceutical, or therapeutic use. Well‐designed preclinical and clinical studies are necessary to provide the evidence required for regulatory approval.

Future research should focus on overcoming these barriers by optimizing, validating, and standardizing the processes and study designs to enhance the reliability and validity of the findings. Moreover, high‐quality studies with larger sample sizes, greater methodological rigor, clinical trials, and comprehensive documentation are needed to confirm the safety and efficacy of nanoemulsions for the skin delivery of EOs.

Additionally, it is crucial to establish criteria to investigate the long‐term effects of EOs nanoemulsion on dermatology, including chronic exposure safety, repeated‐exposure irritation or sensitization, and potential late‐onset immune or inflammatory responses. Long‐term biocompatibility and potential systemic effects should also be evaluated to identify any alterations in the skin barrier or cumulative absorption of the active compounds.

Future studies should focus on selecting EO compositions to define and investigate their efficacy in specific skin conditions, such as surgical wounds, burns, diabetic foot ulcers, dermatitis, eczema, psoriasis, rosacea, mycoses, and acne. These conditions continue to demand new therapeutic options, and many EOs are already used in traditional medicine for their treatment. Exploring these diverse dermatological conditions may help define the true potential of EO nanoemulsions.

## Author Contributions

T.L.M.S. designed the review protocol, conducted the literature search, extracted data, and wrote the article. A.C.M.O.C. conducted the literature search and reviewed data extraction. P.C.F. and F.L.B. supervised the project and critically revised the manuscript. All authors read and approved the manuscript.

## Funding

This work was supported by Coordenação de Aperfeiçoamento de Pessoal de Nível Superior (grant no. 001).

## Conflicts of Interest

The authors declare no conflicts of interest.

## Supporting information


**Data S1:** ptr70184‐sup‐0001‐Supinfo.docx.

## Data Availability

The data that support the findings of this study are available on request from the corresponding author. The data are not publicly available due to privacy or ethical restrictions.
